# Special Issue: Discovery of Novel Antidepressants and Anxiolytics

**DOI:** 10.3390/ph19101541

**Published:** 2026-09-29

**Authors:** Gunnar P. H. Dietz, James Oluwagbamigbe Fajemiroye

**Affiliations:** 1 Georg-August-Universität Göttingen Medical School, D-37075 Göttingen, Germany; 2 Graduate Program in Pharmaceutical Sciences, Evangelical University of Goiás, Anápolis 75080-000, Brazil; 3 Institute of Biological Sciences, Federal University of Goiás, Goiânia 74605-010, Brazil

Figure 1 provides a summary of this special issue.

Besides their debilitating effects on the individual, the socio-economic costs of anxiety and depression are tremendous. The WHO estimates that 12 billion productive workdays are lost annually, translating to about US$1 trillion in lost productivity per year [9]. Thus, higher investment in these conditions is strongly warranted. It has been estimated that scaling up treatment between 2016 and 2030 would require an additional US$147 billion investment, with benefits ranging from 3.3- to 5.7-fold, depending on the country [10].

However, solely investing in established treatments is insufficient. The landmark STAR*D study found that the use of SSRIs only led to remission in 28–33% of depression patients [11]. Of patients with insufficient response, only about one-fourth respond to treatment with other antidepressants [12]. In a more recent review, McIntire et al. claimed that 55% of depressed patients are treatment-resistant, when “a more stringent and multidimensional definition of this condition emphasizing symptomatic remission” is applied [13].

For anxiety disorder, the numbers are not substantially more positive either. An international Delphi consensus concluded that “only 50 to 67% of patients show an adequate clinical response after the first treatment trial. There is, therefore, a pressing need for clinical trials probing novel pharmacological and psychotherapeutic interventions” [14].

Thus, a Special Issue on the discovery of novel antidepressants and anxiolytics is timely and meaningful. The above numbers suggest that the etiology of both psychiatric conditions is highly variable. The term “personalized medicine” as a prevailing research paradigm has at times been asserted in areas where a practically meaningful basis for personalization remained elusive. However, in the context of anxiety and depression, a “one-fits-all” treatment approach may not be feasible, as developmental age, physiological status, presence of other diseases, and substance abuse have to be considered. For instance, different approaches are needed to treat anxiety and depression in children and adolescents [15,16]; older adults [17,18]; pregnant women [19]; people with dementia [20]; Parkinson’s disease patients [21]; cancer patients [22]; cardiovascular disease patients [23]; and people with autism spectrum disorder [24].

The clinical and biological heterogeneity and frequent presence of comorbidities imply that effective disease management, particularly in more severe, persistent, and recurrent cases, requires a multifaceted approach, possibly including pharmacological, psychological, behavioral, physical, or social interventions. For instance, the combination of pharmacotherapy and psychotherapy is more effective in alleviating anxiety than either one alone [25]. Moreover, pharmacologically addressing several signaling pathways may also be a more promising approach than only targeting one specific receptor [7,26].

As shown in Figure 1, this Special Issue addresses neurobiological mechanisms and therapeutic targets, novel drug candidates, and clinical evaluation, with the aim of advancing safer and more effective treatments for anxiety and depression.

## Figures and Tables

**Figure 1:**
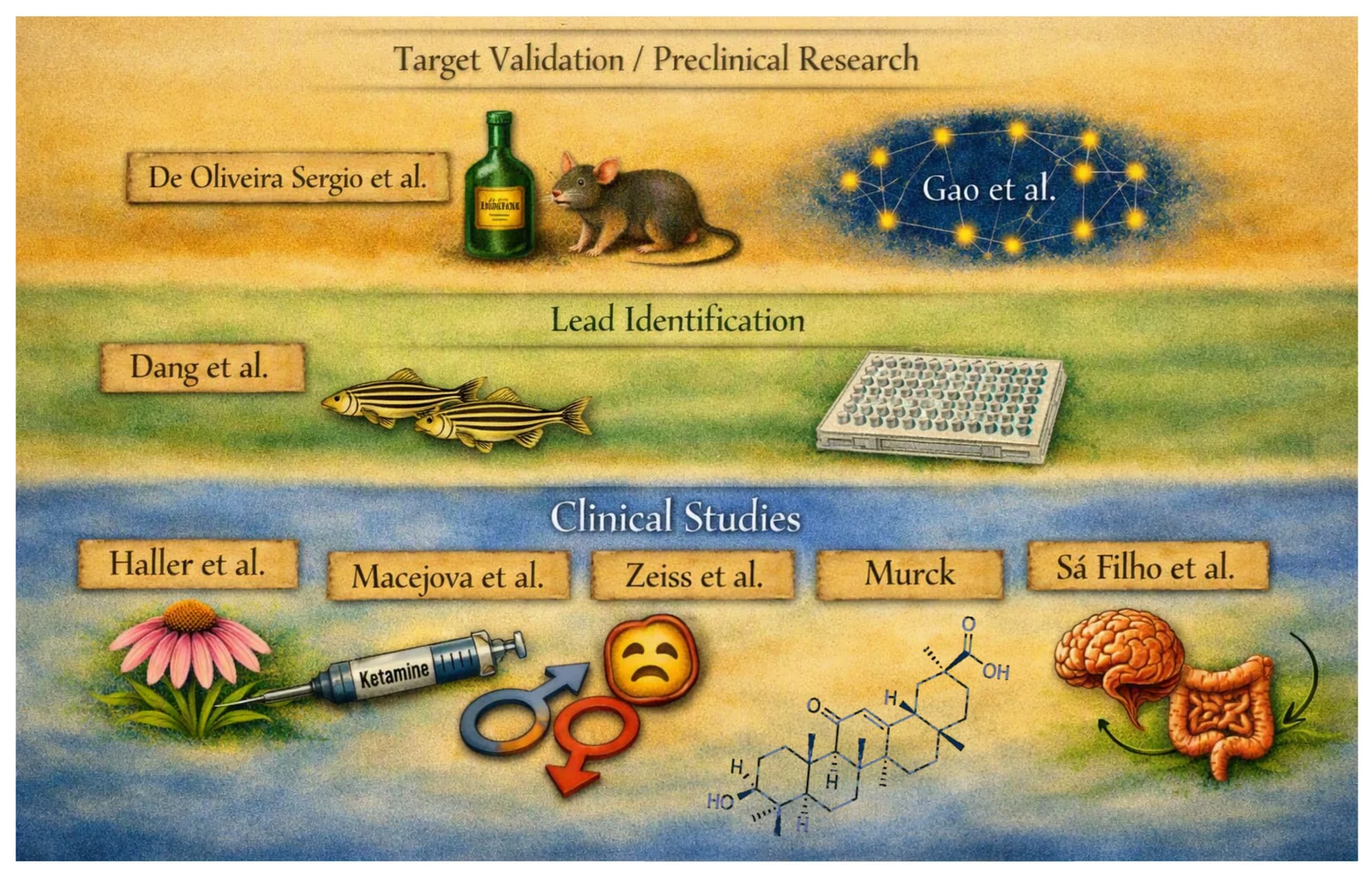
This Special Issue covers a broad range of topics around target validation, lead identification, and clinical studies to develop new treatments for anxiety and depression. De Oliveira Sergio et al. further characterize the role of α1 adrenergic receptors in mediating panic and anxiety-like behaviors in alcohol-dependent rats [1]. A broader, non-hypothesis-driven approach was pursued by Gao et al. to identify potential molecular targets for the treatment of depression [2]. They identified 62 constituents of an herb traditionally used to treat depression in China. Network pharmacology, molecular docking studies, and additional molecular characterization suggested that, for instance, AktI and PIK3R1 interact with various compounds present in the herb examined. A bottleneck in the identification of new chemical entities to treat neurological or psychiatric disease can be the availability of medium- to high-throughput screening assays. In this issue, Dang et al. describe a smart method to screen compounds affecting anxiety behavior in zebrafish [3]. As some of the neuronal signaling mediating depression and anxiety have been preserved in vertebrates, zebrafish are an auspicious system for initial screening approaches. The remaining five papers in this Special Issue provide results from clinical research, including the following: the efficacy or safety of treating depressed or anxious patients using *Echinacea* extract [4]; ketamine or midazolam in adolescent treatment-resistant patients with depression [5]; the effects of enoxolone [6]; the impact of probiotics, prebiotics, and synbiotics [7]; and the unwanted effects of antidepressant use on sexual function [8]. The Figure was generated using Microsoft Copilot in Edge (see Acknowledgements for details).
